# The Utility of the WHO Intrinsic Capacity Screening Tool to Identify Physical and Mental Function Declines

**DOI:** 10.1093/geroni/igab046.710

**Published:** 2021-12-17

**Authors:** Lina Ma, Yaxin Zhang, Pan Liu, Yun Li

**Affiliations:** Xuanwu Hospital, Capital Medical University, National Research Center for Geriatric Medicine, Beijing, Beijing, China (People's Republic)

## Abstract

Background: The disease concept is increasingly being replaced by a functional approach to address the healthcare needs of the older people. WHO proposed the Integrated Care for Older People (ICOPE) screening tool to identify older people with priority conditions associated with declines in intrinsic capacity (IC). Very few evidence on the clinical utility of the ICOPE tool is available. Objectives: To determine if the tool can identify adults with poor physical and mental function. Method: 376 participants aged 50–97 years were included. IC was assessed with the WHO ICOPE screening tool, covering the following five domains: cognitive decline, limited mobility, malnutrition, sensory loss, and depressive symptoms. We assessed the activities of daily living, the Fried frailty phenotype, FRAIL scale, SARC-F scale, MMSE, GDS, social frailty, and quality of life. Peak expiratory flow, bones mineral density, body composition were obtained. Results: 69.1% of the participants showed declines in IC. Participants with declines in IC were older, had more chronic diseases, worse general health, worse physical function as indicated by lower Barthel index, walk speed, grip strength, and physical fatigue, worse mental function indicated by lower MMSE scores, higher GDS scores, more mental fatigue, and worse social function. After adjusting for age, IC was positively correlated with walking speed, resilience score, and MMSE score and negatively correlated with frailty, SARC-F score, IADL score, GDS score, and physical and mental fatigue. Conclusion: The WHO ICOPE screening tool is useful to identify adults with poor physical and mental function in Chinese older adults.

